# Single-Session Anodal tDCS with Small-Size Stimulating Electrodes Over Frontoparietal Superficial Sites Does Not Affect Motor Sequence Learning

**DOI:** 10.3389/fnhum.2017.00153

**Published:** 2017-04-03

**Authors:** Fahimeh Hashemirad, Paul B. Fitzgerald, Maryam Zoghi, Shapour Jaberzadeh

**Affiliations:** ^1^Department of Physiotherapy, School of Primary Health Care, Faculty of Medicine, Nursing and Health Sciences, Monash University, MelbourneVIC, Australia; ^2^Monash Alfred Psychiatry Research Centre, the Alfred and Monash University Central Clinical School, MelbourneVIC, Australia; ^3^Department of Medicine at Royal Melbourne Hospital, the University of Melbourne, MelbourneVIC, Australia

**Keywords:** non-invasive brain stimulation, motor sequence learning, transfer of learning, transcranial magnetic stimulation, primary motor cortex, dorsolateral prefrontal cortex, posterior parietal cortex

## Abstract

Due to the potential of anodal transcranial direct current stimulation (a-tDCS) for enhancement of fine sequenced movements and increasing interest in achieving high level of fine movements in the trained and untrained hands especially at initial stage of learning, we designed this study to investigate whether the application of single-session a-tDCS with small-size stimulating electrodes over FPN sites, such as dorsolateral prefrontal cortex (DLPFC), primary motor cortex (M1) or posterior parietal cortex (PPC) could enhance sequence learning with the trained hand and these effects are transferred into the untrained hand or not. A total of 51 right-handed healthy participants were randomly assigned to one of the four stimulation groups: a-tDCS of left M1, DLPFC, PPC, or sham. Stimulation was applied for 20 min during a sequential visual isometric pinch task (SVIPT). Eight blocks of training using SVIPT were completed with the right hand during stimulation. Two blocks of sequence training with each hand were performed by participants as assessment blocks at three time points: baseline, 15 min and one day following the intervention. Behavioral outcomes including movement time, error rate and skill were assessed in all assessment blocks across three time points. We also measured corticospinal excitability, short-interval intracortical inhibition, and intracortical facilitation using single- and paired-pulse transcranial magnetic stimulation. The results indicated that the behavioral outcomes were significantly improved with the right trained hand, but this learning effect was not modulated by a-tDCS with small-size stimulating electrodes over the FPN. Transfer of learning into the untrained hand was observed in all four groups for movement time but not for the error rate or skill. Our results suggest that sequential learning in SVIPT and its transfer into the untrained hand were not sensitive to a single-session a-tDCS with small-size stimulating electrodes over left M1, DLPFC or PPC in young healthy participants.

## Introduction

Learning sequences in fine movements plays a crucial role in everyday life and requires a strong coordination between visual and motor cortex. Finding novel techniques to improve rehabilitation in fine movements in the trained hand as well as transfer of learning into the untrained hand would implicate for patients who struggle with fine motor tasks, such as those with stroke or Parkinson’s disease. Recently, anodal transcranial direct current stimulation (a-tDCS), which modulate brain activity, has allowed direct investigation of the role of specific areas of the brain during different stages of sequence learning (Nitsche et al., 2003; Fregni et al., 2005; Reis et al., 2009; Schambra et al., 2011; Zaehle et al., 2011; Javadi and Walsh, 2012; Pope and Miall, 2012; Hoy et al., 2013; Saucedo Marquez et al., 2013; Weiss et al., 2013; Convento et al., 2014; Waters-Metenier et al., 2014; Reis et al., 2015; Rivera-Urbina et al., 2015).

A large body of neuroimaging evidence has revealed that sequence learning is mediated by frontoparietal network (FPN) superficial sites including dorsolateral prefrontal cortex (DLPFC) (Jenkins et al., 1994; Sakai et al., 1998; Miller and Cohen, 2001; Hasan et al., 2013), the primary motor cortex (M1) (Grafton et al., 1995; Karni et al., 1995; Hazeltine et al., 1997; Rioult-Pedotti et al., 2000) and posterior parietal cortex (PPC) (Jenkins et al., 1994; Sakai et al., 1998). The contribution of specific areas of the FPN may change across sequence learning depends on the stage of learning (Karni et al., 1998; Doyon and Ungerleider, 2002; Dayan and Cohen, 2011). M1 known to play an important role in acquisition and consolidation of movements, while rapid improvements gained over the course of a single training session (fast stage of learning) are more associated with the activity of DLPFC or PPC (Sakai et al., 1998; Koch et al., 2008a,b).

Although there is a large number of studies providing evidence for efficacy of multiple-sessions a-tDCS over M1(which links to slow stage of learning) (Reis et al., 2009; Schambra et al., 2011; Saucedo Marquez et al., 2013; Waters-Metenier et al., 2014; Hashemirad et al., 2016), the efficacy of single-session of M1 a-tDCS remains controversial.

Exploring single-session a-tDCS effects over other areas of the FPN, such as DLPFC or PPC, which are more associated with initial stages of learning are necessary to be investigated to determine the optimum stimulation sites to influence sequence learning. In this study, we applied single-session a-tDCS over three different areas of the brain (M1, DLPFC, or PPC) during a sequential visual isometric pinch task (SVIPT) in order to assess the effects of a-tDCS on a fine-motor control task in young healthy individuals at the early stage of learning. We also examined the effects of a-tDCS on transfer learning into the untrained hand by quantifying generalization behavioral outcomes into the untrained hand. To evaluate possible underlying mechanisms which are responsible for the effects of a-tDCS during SVIPT, we also measured changes in M1 corticospinal excitability (CSE), short-interval intracortical inhibition (SICI) and intracortical facilitation (ICF) using a single- or paired-pulse transcranial magnetic stimulation (TMS).

To the best of our knowledge, this is the first study to verify the effects of single-session a-tDCS over the FPN sites on implicit motor sequence learning and transfer learning into untrained hand using SVIPT. The aims of this study were to investigate: (1) the effects of a-tDCS of M1, DLPFC, or PPC on cortical and behavioral changes during motor sequence learning using SVIPT, (2) the correlation between behavioral and cortical effects, and (3) whether the acquired behavioral changes during the training are transferable to the untrained hand.

## Materials and Methods

### Study Design

This study was a parallel randomized single-blind sham-controlled study where each participant took part in one of the four stimulation conditions.

### Participants

Fifty-one healthy participants (36 females, 15 males; age between 18 and 40 years old with *mean ± SD*; 25.82 ± 6.14 were randomly assigned to one of the four stimulation groups: (1) a-tDCS of left M1, (2) a-tDCS of left DLPFC, (3) a-tDCS of left PPC, (4) sham a-tDCS. All participants were right-handed based on the Edinburgh Handedness Inventory (Oldfield, 1971) (Laterality index: 78.83% ± 20.98). Exclusion criteria for participation in the experiments were: (1) having contraindications to be assessed by TMS or for receiving tDCS, e.g., having a seizure or with the family, having any metal in their head, severe headaches and pregnancy, (2) current usage of any medicine which could affect the brain excitability, motor learning or cognition, (3) history of neurological or psychiatric diseases, (4) significant experience with musical instruments or computer games (more than 5 hours of practice in a day or 1000 h of practice during the last six months before the study), (5) disability in fingers, hand or wrist, (6) age above 40 years or less than 18 years. All participants were naive to the purpose of the experiments. All tests were conducted between 8 am and 4 pm. To control for the effect of female hormonal fluctuation on the size of MEPs, the experimental sessions were carried out between the 7th and 23th day of women’s menstrual cycles. Information about sleep hours, quality of sleep and experience with computer games were also obtained through a brief questionnaire. This study was carried out in accordance with the recommendations of the Human Ethics Committee at Monash University with written informed consent from all subjects. All subjects gave written informed consent in accordance with the Declaration of Helsinki. The protocol was approved by the Human Ethics Committee at Monash University.

### TMS Measurement

A MagPro R30 stimulator (MagVenture) with a butterfly coil (MC-B70) and dimensions (169 × 112 × 16/33 mm) was used to induce motor-evoked potentials (MEPs) from the right first dorsal interosseous (FDI) muscles. The coil was placed over the left M1 region with a posterior–anterior orientation and set at an angle of 45° to the midline. The area of stimulation with largest MEP responses was defined as the hotspot and marked on the scalp to ensure consistency of coil placement throughout the experiment. Resting motor threshold (RMT) was defined as the minimal stimulator output needed to elicit three out of six MEPs with minimum amplitude of 50–100 μV in a relaxed FDI muscle (Rossini and Rossi, 1998). All raw EMG signals, were amplified, filtered (20 Hz–10 kHz) and recorded with a PC running a commercially available data acquisition and automated-analysis package (PowerLab^TM^ ADInstrument 4/35 with LabChart^TM^, Australia) for offline analysis.

### Single-Pulse TMS

Single-pulse TMS (MagPro R30 stimulator) was used over the left M1 in order to record MEPs from the right FDI muscle. Test TMS intensity was adjusted to produce a test MEP of about 1mV in FDI muscle at rest. Twenty single-pulse were delivered with 10 s inter pulse interval and 20 MEPs were recorded from the right FDI muscle. Average peak-to-peak amplitudes of 20 MEPs were calculated for each time point (Baseline, post 15 min and post 24 h) to assess CSE of M1.

### Paired-Pulse TMS

Paired-pulse TMS (MagPro R30 stimulator) was used to evaluate SICI and ICF in M1. In this paradigm, a sub threshold conditioning stimulus was followed by a supra threshold test stimulus (Kujirai et al., 1993). The amplitude of the conditioning stimulus was set to 80% of the RMT and unconditioned stimulus or test stimulus was adjusted at 1mV. Paired-pulse TMS was delivered randomly in a block of 40 trials with inter-stimulus intervals (ISI) of 3 or 10 ms, respectively. MEP areas were quantified for conditioned and unconditioned stimuli using a custom designed macro in Power Lab 4/35 software. The size of the conditioned MEPs was expressed as a percentage of unconditioned test MEPs at baseline. Test intensity was adjusted to elicit an unconditioned MEP with peak-to-peak amplitudes of 1 mV at the following day.

### Transcranial Direct Current Stimulation (tDCS)

A commercially available stimulator (Intelect Advanced Therapy System, Chattanooga, TN, USA) was used to deliver direct current with intensity of 0.3 mA for 20 min through a pair saline-soaked rectangular sponge surface electrodes. The size of active and return electrodes were 2 × 1.5 (3 cm^2^) and 4 × 3 (12 cm^2^), respectively. The small size of electrodes yield a highly focused direct current over the target areas, which enabled us to stimulate the target areas without stimulating nearby areas (Nitsche et al., 2007; Faria et al., 2011; Vaseghi et al., 2015a,b). In this study, we adjusted the current intensity for the small electrode size (3 cm^2^) by keeping the current density (0.1 mA/cm^2^) in a safe range (Nitsche and Paulus, 2000; Poreisz et al., 2007), to modulate the excitability of neurons in the target area (Bastani and Jaberzadeh, 2013a,b; Vaseghi et al., 2015a,b). Therefore, the active electrode with size of 3 cm^2^ was placed over the target areas (left M1, DLPFC, or PPC) and the return electrode (12 cm^2^) was fixed over the contralateral supraorbital region. For the sham group, the active electrode randomly was placed over the three different stimulation areas (M1, DLPFC, or PPC). The distribution for the stimulation conditions was randomly balanced across participants. The current was ramped up to 0.3 mA and then ramped down so that participants felt an initial sensation for 30 s of stimulation.

The locations of M1 was identified using TMS, the location of DLPFC or PPC were determined using the international 10–20 system (Steinmetz et al., 1989). Therefore, the stimulating electrodes for DLPFC or PPC were placed over F3 and P3, respectively. participants were asked to report tDCS side effects such as itching, tingling, burning sensations, headache, pain, and any other sensations (Poreisz et al., 2007). All participants rated the presence and severity of these side effects using numeric analog scales (NAS) (e.g., 0 = no feeling to 10 = worst feeling imaginable). To check the blinding integrity, after completion of the stimulation session, participants were asked to indicate if they thought they had received active or sham stimulation.

### Apparatus and Task

**FIGURE 1:**
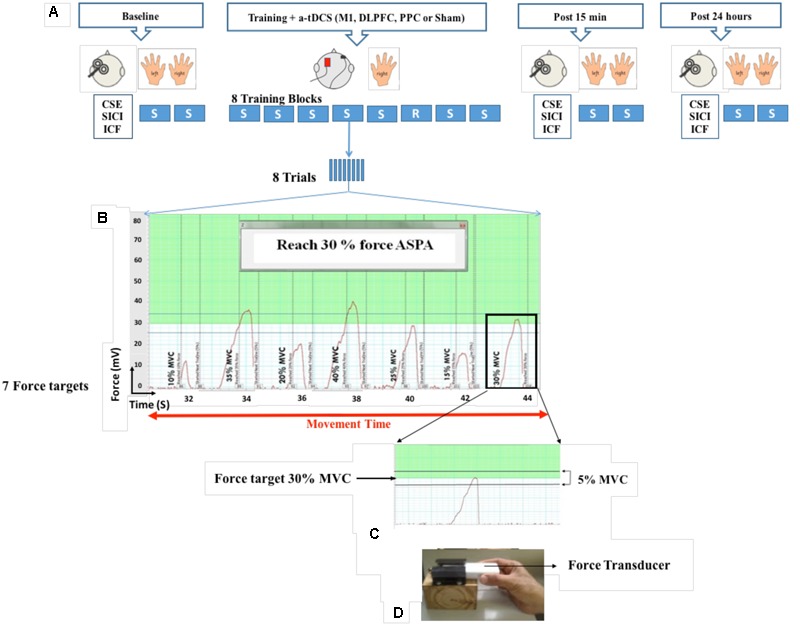
**Experimental set-up.** Participants held a force transducer between their thumb and index finger and altered their precision force on the force transducer to move a cursor on the computer screen to reach different target forces. During eight blocks of training, a-tDCS (left M1, DLPFC, PPC, and sham) were applied over the left hemisphere contralateral to the performed hand. Each block consisted of eight trials and each trial included seven target forces from 10 to 30 % of MVC which appeared on the computer screen. Cortical and behavioral changes were assessed over three time points at baseline, post 15 min and post 24 h after intervention **(A,B)**. Participants were required to squeeze the force transducer to reach the target force in a range of 5% below or above the target force **(C,D)**. SVIPT: Sequential visual isometric pinch task, A-tDCS: Anodal transcranial direct current stimulation, M1, Primary motor cortex; DLPFC, Dorsolateral prefrontal cortex; PPC, Posterior parietal cortex; S, Sequence block; R, Random block; CSE, Corticospinal excitability; SICI, Short-interval intracortical inhibition; ICF, intra-cortical facilitation (ICF); maximum voluntary contraction (MVC).

A force transducer (AD instrument MLT004/ST, NSW, Australia) was used for induction of SVIPT in this study. SVIPT is a pinch force task in which participants were asked to squeeze the force transducer between their thumb and index finger to move a cursor upward on the computer screen to meet different target forces (**Figure 1**). At the beginning of each experiment, maximum voluntary contraction (MVC) was individually determined for each participant. Two trials were then given as familiarization. After familiarization, two sequence blocks were randomly performed as baseline measurement with each hand. Each sequence block consisted of eight trials and each trial included seven target forces which appeared in a sequence order (10, 35, 20, 40, 25, 15, and 30% MVC) on the computer screen. The inter-trial interval was set at 1 s. Each target force was only presented once in each trial. The level of each target force was determined by a green line or a numerical number in an indicator box on the computer screen. Participants were instructed to squeeze the force transducer to reach the target force in a range of 5% below or above the target force. More or less than this range was considered as an over- or under-shoot error. During training, each participant completed eight blocks of the same sequence order with dominant hand, except for the block 6 which was set in a random order. Inter-block interval was set at 1 min. Each participant completed the training in approximately 20–25 min. Participants were received no feedback during training. They were also not aware of the sequential order of the target forces in each trial. To make sure implicit learning, they were asked to recall target forces to determine the amount of their awareness. If they could recall more than three consecutive target forces, leaning was considered as explicit and their data were excluded from analysis. Fifteen min after completion of the training, participants completed two blocks as a post-test with each hand randomly. One day after training, two blocks were repeated as a retention test with each hand. The number of trials, as well as sequence order of target forces, were the same in the both training and assessment blocks.

The following behavioral outcomes were measured in each assessment block:

### Movement Time

Movement time in each trial, was defined as the time from movement onset for the first target to cessation of movement after the final target as shown in **Figure 1B**. The mean movement time for eight trials was taken as the movement time for the given block (Reis et al., 2009).

### Error Rate

The error rate was calculated as the proportion of the trials with at least one over- or undershoot (Reis et al., 2009). Participants needed to meet all seven targets in each trial correctly to get the accuracy of that trial.

### Skill

Skill, which is defined as a combination of both parameters of movement time and error rate, represents changes in the speed-accuracy trade-off. This variable was obtained from the following formula suggested by Reis et al. (2009).

(1) $$\text{Skill}\;\text{=}\;\frac{\text{1}\;\text{-}\;\text{error}\;\text{rate}}{\text{error}\;\text{rate}\;[\text{ln}\;{\left(\text{movement}\;\text{time}\right)}^{\text{5}.\text{424}}]}.$$

### Experimental Procedure

In each experiment, the same procedure was followed: (1) baseline measurements (TMS and SVIPT), (2) training paired with anodal/sham tDCS stimulation, (3) post measurements (TMS and SVIPT) after 15 min and (4) post measurements (TMS and SVIPT) after 24 h (**Figure 1**). To decrease the effects of order, TMS and SVIPT was randomized for each assessment, as was the choice of the performing hand.

### Data Analysis

Kolmogorov–Smirnov (K–S) test was used to assess the normality of data. For all normal distributed variables, a mixed-design ANOVA (Repeated-measure) with the factor of Time (baseline, post 15 min after and post 24 h) as a within-subjects factor and factor of Group (a-tDCS of M1, DLPFC, PPC, or sham) as between-subjects factor was conducted to assess the effects of a-tDCS on motor sequence learning among the four groups over time. This analysis was separately applied for assessment blocks with trained and untrained hands. A Greenhouse–Geisser test was used in order to correct non-sphericity if the assumption of Mauchly’s test of sphericity was violated. *Post hoc* tests with Bonferroni correction were performed as appropriate to determine where differences occurred.

For non-normally distributed data, log transformation was performed in order to achieve normal distributions of the data. After the transformation, if normal distribution were not corrected and the skewness of the log data were still more than one, non-parametric tests were conducted. The Friedman two-way analysis of variance by ranks was used to assess differences in mean rank of non-parametric variables across three time points. A K-independent method by median test was conducted to evaluate whether the groups differed in their median or not. A Kruskal–Wallis test one-way analysis by rank was used if median test was not computed when all data were equal or less than median. Equality of deviation of mean rank among the four groups as an assumption for Kruskal–Wallis method was tested by Levin’s test of non-parametric variables. Bonferroni correction was used for correction of multiples groups, if differences between groups was determined.

Pearson correlation was conducted to investigate relationship between cortical and behavioral outcomes. SPSS (version 20) and MATLAB (R2014a) were used to analysis the data in this study. Statistical significance was set at *p* < 0.05.

## Results

Of the 51 participants enrolled in this study, three subjects were excluded because they could not perform the SVIPT task as instructed.

As shown in **Table 1**, there were no significant difference in participants’ characteristics such as age, right-handedness, MVC, and also some other parametric variables including experience with computer games, sleep hours, sleep quality, attention during task, fatigue, and sequence awareness (*p* > 0.05).

**Table 1:** Participants’ characteristic in the four experimental groups.

	Group M1	DLPFC	PPC	Sham	ANOVA	
	Mean ± SD	Mean ± SD	Mean ± SD	Mean ± SD	*F*	*P*
Number (Female/Male)	12 (8/4)	12 (9/3)	12 (9/3)	12 (8/4)		
Age	27.8 ± 5.8	25.1 ± 5.8	24.8 ± 5.9	25.5 ± 7.1	1.52	0.22
Handedness	75.5 ± 27.7	82.4 ± 15.6	77 ± 19.6	85.7 ± 11.4	0.89	0.45
MVC	61.4 ± 26.2	68.7 ± 22.1	61.5 ± 18.6	70.1 ± 26.3	1.46	0.24
Computer game (Hour in a day)	0.78 ± 1.39	0.2 ± 0.44	0.8 ± 1.3	0. 5 ± 0.57	0.347	0.79
Sleep hour day1	7.3 ± 1.55	6.8 ± 0.83	6.8 ± 1.24	6.8 ± 1.83	0.68	0.57
Sleep quality day1	7.8 ± 1.74	7.3 ± 1.59	8.13 ± 1.12	7.1 ± 1.83	0.68	0.57
Attention day1	8 ± 1.04	8.13 ± 0.99	7.8 ± 1.12	7.8 ± 1.06	0.1	0.954
Fatigue day1	1.67 ± 1.92	0.75 ± 2.12	0.25 ± 0.707	0.43 ± 0.787	1.58	0.21
Sleep hour day2	7.6 ± 1.5	7.5 ± 1.5	7.4 ± 0.54	7.3 ± 2.05	0.064	0.97
Sleep quality day2	8.1 ± 1. 4	8.1 ± 0.75	7.6 ± 1.3	8.5 ± 0.57	0.45	0.71
Attention day2	9.06 ± 0.63	8.5 ± 1.02	8.6 ± 0.54	8.2 ± 0.5	1.33	0.29
Fatigue day2	0	0.67 ± 1.03	1.2 ± 1.7	0	2.09	0.13
Awareness	0.88 ± 1.64	0.4 ± 0.89	0.4 ± 0.54	0.2 ± 0.44	0.43	0.72

There were also no significant differences in cortical outcome measures, including CSE (*p* = 0.82), SICI (*p* = 0.32) and ICF (*p* = 0.87) or behavioral outcomes including movement time (*p* = 0.52), error rate (*p* = 0.64), and skill (*p* = 0.49) among the four groups at the baseline.

There were no significant differences between participants’ feeling in all four condition measurements (Supplementary Table S1). Blinding integrity was intact because participants were not able to determine active versus sham a-tDCS in either group based on the results obtained from Pearson’s chi-square [χ2 (4, *n* = 48) = 1.33, *P* = 0.24].

### Cortical Outcome Measures

Resting motor threshold and test intensity (mean ± SEM) are reported in **Table 2**, for all four groups at each experimental session. The results of one-way ANOVA showed no significant differences in RMT [*F*(3,44) = 1.14, *p* = 0.34] or test intensity [*F*(3,44) = 1.45; *p* = 0.23] at baseline among the groups. In addition, no significant difference was found for either RMT [*F*(1,44) = 3.4, *p* = 0.072] or test intensity [*F*(1,44) = 0.024, *p* = 0.87] between the two experimental sessions.

**Table 2:** Mean of resting motor threshold (RMT) and test intensity as % of maximum stimulator output at the two experimental sessions.

Stimulation groups	RMT		Test intensity	
	Session 1	Session 2	Session 1	Session 2
M1	34.5% ± 1.93	34.5% ± 1.83	47.08% ± 2.77	46.08 % ± 2.85
DLPFC	33% ± 1.17	32.4% ± 1.27	43.7% ± 1.58	42.9 % ± 1.28
PPC	37.3% ± 1.65	36.8% ± 1.6	50.9% ± 2.43	51.9% ± 1.95
Sham	36.5% ± 2.4	35.7% ± 2.06	48.4% ± 2.88	49.5% ± 2.57

*Data are presented as mean ± SEM.*

### Effects of a-tDCS and Training on CSE

**FIGURE 2:**
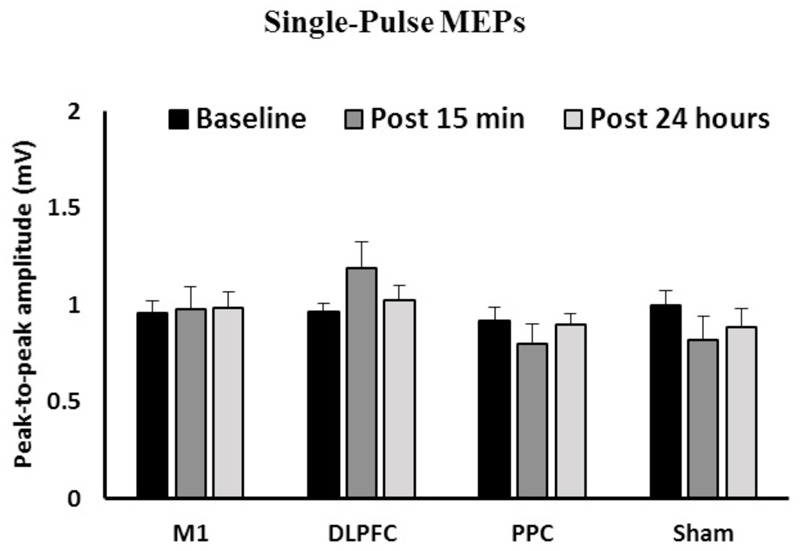
**The mean of peak-to-peak amplitudes of MEPs in the right FDI among the four groups over three time points.** No significant difference was found in the main factors of Time or Group. Data are presented at Mean ± SEM.

**Figure 2** shows the mean peak-to-peak amplitude of MEPs before and after interventions over three time points (baseline, post 15 min and post 24 h) in all four groups. The results of ANOVA showed no main effects of Time [*F*(1.58,67.9) = 0.031, *p* = 0.94] or Group [*F*(3,43) = 1.41, *p* = 0.25]. The interaction between Time and Group [*F*(4.73,67.9) = 1.55, *p* = 0.18] on the size of the MEPs was not significant.

### Effects of a-tDCS and Training on SICI

**FIGURE 3:**
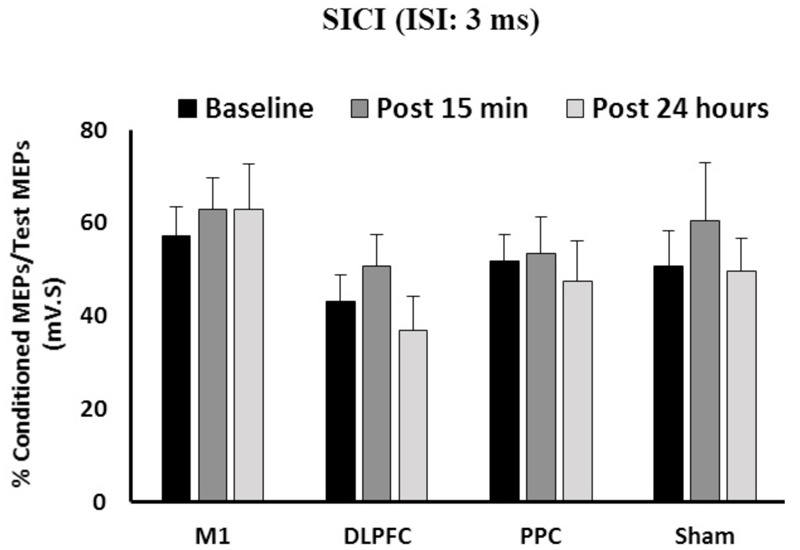
**The mean of SICI in M1 after a-tDCS stimulation among the four condition groups over three time points.** No significant differences were found in main factors of Time or Group. Data are presented at Mean ± SEM.

The results of a mixed-design ANOVA showed that a-tDCS delivered concurrently with training using SVIPT did not modulate SICI across the three time points [*F*(2,86) = 1.58, *p* = 0.21] (**Figure 3**). Main effects of Group was not significant for SICI [*F*(3,43) = 1.45, *p* = 0.24]. In addition, the interaction between Time and Group on SICI was not significant [*F*(6,86) = 0.31, *p* = 0.93].

### Effects of a-tDCS and Training on ICF

**FIGURE 4:**
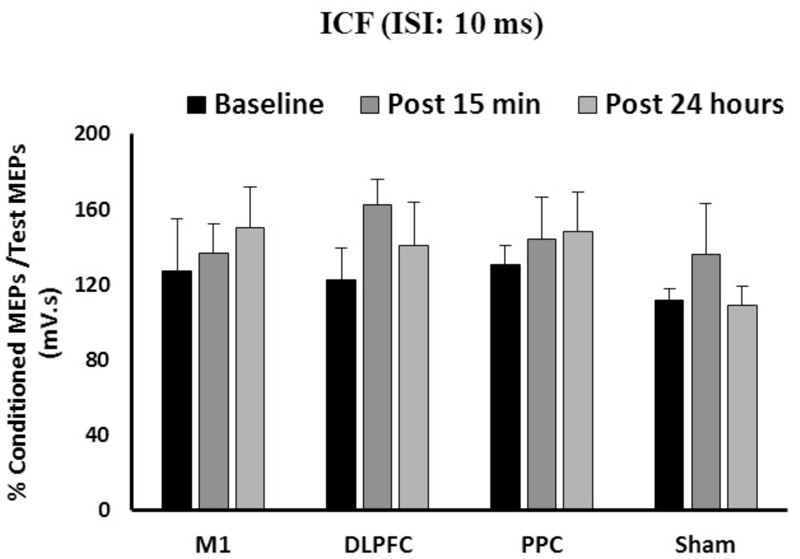
**The mean of ICF in M1 after a-tDCS stimulation among four condition groups over three time points.** No significant differences were found in main factors of Time or Group. Data are presented at Mean ± SEM.

The results of a mixed-design ANOVA showed the main effects of Time [*F*(2, 86) = 1.82, *p* = 0.16] or Group [*F*(3,43) = 0.61, *p* = 0.6] was not significant on ICF (**Figure 4**). The interaction between Group and Time was not significant either on ICF [*F*(6,86) = 0.43, *p* = 0.85].

### Behavioral Outcome Measures

Movement time was normally distributed so we conducted a mixed-design ANOVA to investigate the effects of interventions on this variable in both the trained and untrained hands. In contrast, the error rate and skill were non-normally distributed. Since their normality were not corrected using log transformation, we conducted nonparametric tests on these variables to test the effects of intervention on these variables with both the trained and untrained hands.

### Movement Time

#### Trained Hand

**FIGURE 5:**
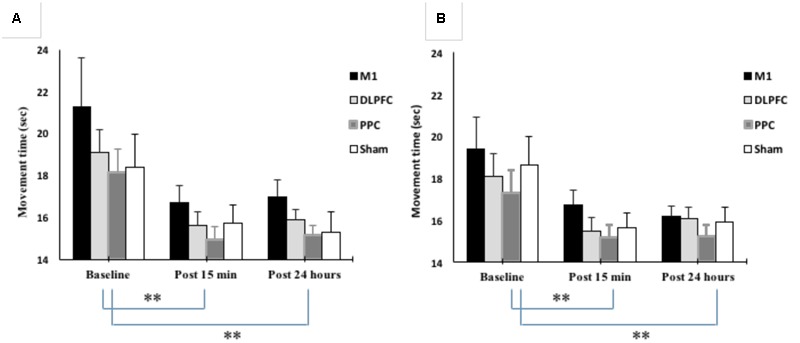
**Changes in movement time in blocks of right trained hand (A)** and left untrained hand **(B)**. The results showed significant improvement in movement times for all four stimulation groups over three time points. ^∗∗^*P* < 0.01.

Mean movement time was decreased from 19.2 ± 5.6 at baseline to 15.7 ± 2.5 at post 15 min and 15.8 ± 2.5 at post 24 h after intervention. The results of mixed-design ANOVA showed significant improvement in movement time with the right trained hand over the three time points [*F*(1.15,50.6) = 20.1, *p* < 0.001] (**Figure 5A**). *Post hoc* analysis with Bonferoni correction showed that the movement time significantly decreased 15 min and 24 h after intervention compared to baseline (*p* < 0.001). However, there was no significant difference between two post-tests (*p* = 1).

The main effect of the stimulation group was not significant for the trained right hand [*F*(3, 44) = 1.302, *p* = 0.28]. There was also no interaction between Group and Time [*F*(3.45, 50.6) = 0.24, *p* = 0.89].

#### Untrained Hand

As shown in **Figure 5B**, movement time significantly improved in the left untrained hand [*F*(1.26,55.5) = 22.4, *p* < 0.001]. The results of *post hoc* analysis showed that the movement time significantly decreased at post 15 min (15.7 ± 2.29) compared to baseline (18.3 ± 4.18) (*p* < 0.001). This reduction remained in the following day (15.8 ± 1.94) and significantly different from baseline (*p* < 0.001). However, there was no significant difference between post-tests (*p* = 1).

The main effect of stimulation groups for the left hand was not significant [*F*(3,44) = 0.79, *p* = 0.503]. Interaction between Group and Time was also not significant [*F*(3.78,55.5) = 0.27, *p* = 0.88].

### Error Rate

The minimum, maximum, and mean rank as well as median of the error rate are represented in **Table 3** for both the trained and untrained hands over three time points.

**Table 3:** The minimum, maximum, mean rank, and median of the error rate in assessment blocks performed with trained (right) or untrained (left) hand at three time points.

Error rate *N* = 48	Group	Min–Max/Mean rank			Median		
		Baseline	Post 15 min	Post 24 h	Baseline	Post 15 min	Post 24 h
Trained (right)	M1	2.5–32/23.6	8–38/28.6	2.5–39/28.5	1	0.87	0.87
	DLPFC	5.5–32/ 24.6	2–38/18.21	2.5–39/19.3			
	PPC	5.5–32/26.3	5–38/29	2.5–39/26.8			
	Sham	1–32/23.2	2–38/22.1	2.5–39/23.2			
Untrained (left)	M1	4.5–34/23.8	5–37/29.5	7–36/24.2	1	0.87	1
	DLPFC	1–34/19.1	1–37/23.2	1–36/22.5			
	PPC	4.5–34/26.2	5.5–37/25.8	5–36/29.2			
	Sham	13–34/28.7	2.5–37/19.3	3–36/21.9			

#### Trained Hand

**FIGURE 6:**
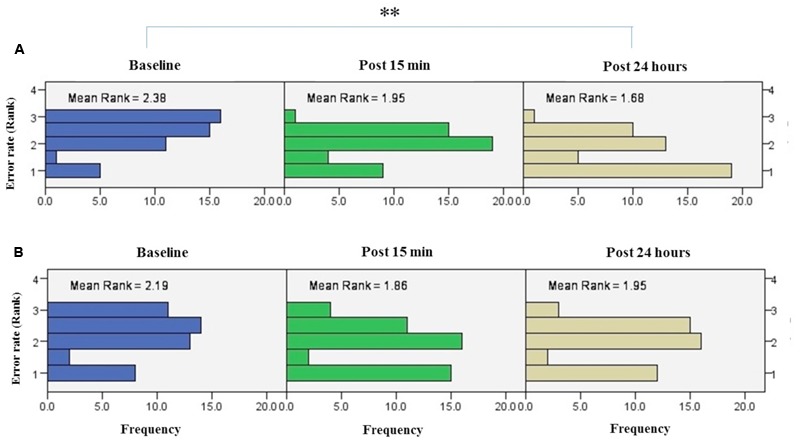
**Results of Friedman test and distribution of error rate by rank in both right trained hand (A)**, left untrained hand **(B)** over three time points. Asterisks indicate significant differences in mean rank across time points. ^∗∗^*P* < 0.01.

Friedman’s test showed a statistically significant decrease in the error rate for the right hand [χ2(2, *n* = 48) = 17.9, *p* < 0.001] (**Figure 6A**). The mean rank of the error rate decreased from 2.38 at baseline to 1.95 at post 15 min and to 1.68 at post 24 h. *Post hoc* analysis with a Bonferroni correction showed that there was only a significant difference between baseline and post 24 h (*p* = 0.002). No significant difference was found between baseline and post 15 min (*p* = 0.109) or two post-tests (*p* = 0.554).

**FIGURE 7:**
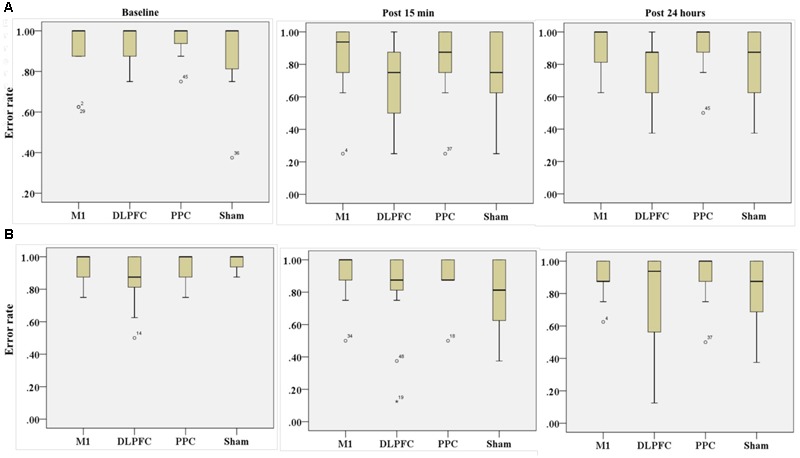
**Effects of a-tDCS and training on error rate among the four stimulation groups in the right trained hand (A)** and left untrained hand **(B)** at three time points. No significant effects were found between all stimulation conditions.

The results of K-independent samples showed that there were no significant differences among the four groups at baseline [χ^2^(3, *n* = 48) = 0.52, *p* = 0.91], post 15 min [χ^2^(3, *n* = 48) = 5.67, *p* = 0.12] or post 24 h after intervention [χ^2^(3, *n* = 48) = 3.2, *p* = 0.36] (**Figure 7A**). Therefore, a-tDCS had no site-specific effects on error rate at any stimulation groups over times.

#### Untrained Hand

Friedman’s test indicated no significant decrease in error rate for assessment blocks which performed with the left untrained hand over the three time points [χ^2^(2, *n* = 48) = 4.01, *p* = 0.134] (**Figure 6B**).

Results of K-independent samples showed no significant changes in error rate of the left hand across the groups over the three time points (**Figure 7B**). The effects of a-tDCS and training were the same across the four groups at baseline [χ^2^(3, *n* = 48) = 4.08, *p* = 0.25], post 15 min [χ^2^(3, *n* = 48) = 3.02, *p* = 0.38] as well as post 24 h after intervention [χ^2^(3, *n* = 48) = 2.36, *p* = 0.5].

### Skill

**Table 4** represents the minimum, maximum and mean rank as well as median of skill for both the trained and untrained hands over three time points.

**Table 4:** The minimum, maximum, mean rank and median of skill in assessment blocks performed with trained (right) and untrained (left) hand at three time points.

Skill	Group	Min–Max/Mean rank			Median		
		Baseline	Post 15 min	Post 24 hours	Baseline	Post 15 min	Post 24 hours
Trained (right)	M1	17–47/25.7	11–40/19.8	9.5–45/20.7	0E-7	0.0092	0.01
	DLPFC	17–42/24	11–48/31.1	9.5–46/29.4			
	PPC	17–43/22.5	11–44/20.1	9.5–48/21.8			
	Sham	17–48/25.6	11–46/26.8	9.5–47/25.9			
Untrained (left)	M1	15–46/25	11.5–46/19.4	13–42/24.5	0E-7	0.0091	0E-7
	DLPFC	15–48/29.9	11.5–48/25.3	13–48/26.5			
	PPC	15–44/23	11.5–43/23	13–44/19.7			
	Sham	15–41/19.9	11.5–46/30.1	13–45/27.1			

#### Trained Hand

**FIGURE 8:**
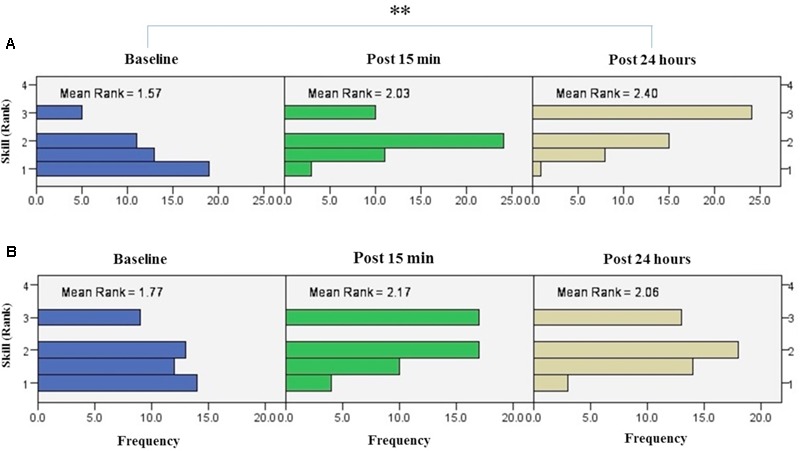
**Results of Friedman test and distribution of skill by rank in blocks of the right hand (A)** and left hand **(B)** over three time points. Asterisks indicate significant differences in mean rank across time points. ^∗∗^*P* < 0.01.

The results of Friedman test showed a significant increase in mean rank of skill with the right trained hand [χ^2^(2, *n* = 48) = 22.3, *p* < 0.001] (**Figure 8A**). The mean rank of skill increased from baseline (1.57) to post 15 min (2.03) and post 24 h (2.4). *Post hoc* analysis with Bonferroni correction showed that skill significantly improved at post 24 h after intervention compared to the baseline measurement (*p* < 0.001) but this increase was not significant between baseline and 15 min after intervention (*p* = 0.074) or between two post-tests (*p* = 0.22).

**FIGURE 9:**
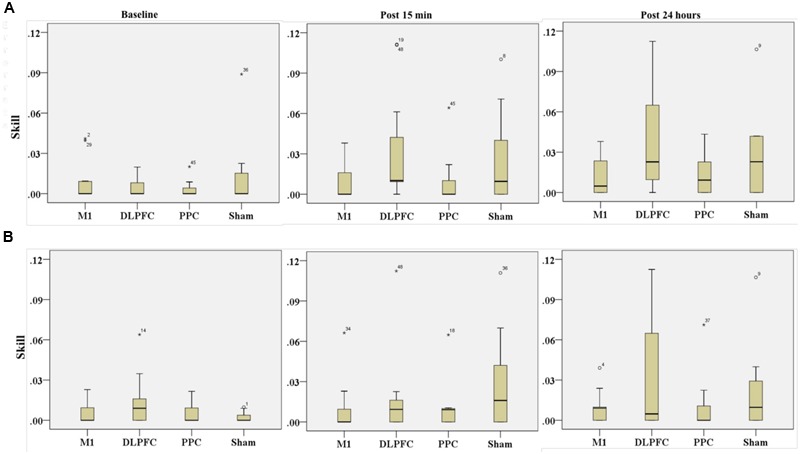
**Effects of a-tDCS and training on skill among the four stimulation groups in the right trained hand (A)** and left untrained hand **(B)** at three time points. No significant effects were found between all stimulation conditions.

The results of K-independent test revealed no significant differences in skill across the four groups in assessment blocks with the dominant right hand (**Figure 9A**). The effects of a-tDCS and training were the same across the four groups at baseline [χ^2^(3, *n* = 48) = 0.291, *p* = 0.962], post 15 min [χ^2^(3, *n* = 48) = 6, *p* = 0.112] as well as post 24 h [χ^2^(3, *n* = 48) = 3.33, *p* = 0.343].

#### Untrained Hand

The results of Friedman’s test in the assessment block with the left untrained hand revealed a trend of improvement in skill over time [χ^2^(2, *n* = 48) = 5.62, *p* = 0.06] (**Figure 8B**).

Results of K-independent samples showed no significant changes in the error rate of left hand across the groups over the three time points (**Figure 9B**). The effects of a-tDCS and training were the same across the four groups at baseline [χ^2^(3, *n* = 48) = 3.04, *p* = 0.384], post 15 min [χ^2^(3, *n* = 48) = 4.66, *p* = 0.198] as well as post 24 h after interventions [χ^2^(3, *n* = 48) = 3.59, *p* = 0.309].

### Correlation between Cortical and Behavioral Outcomes

A Pearson correlation test was conducted to determine the relationship between cortical and behavioral outcomes for two experimental sessions. No correlations were found between cortical and behavioral outcomes except for movement time and ICF, which showed a low inverse relationship at the second session (*r* = –0.41, *p* = 0.003) (Supplementary Table S2). This result indicates that decrease in movement time for performing SVIPT was correlated by increase in facilitation of interneurons of M1 at one day after intervention.

## Discussion

In this study, we applied single-session a-tDCS with small-size stimulating electrodes over M1, DLPFC, PPC, or sham during training with SVIPT in young healthy participants. The effects were investigated on both cortical (CSE, SICI, and ICF) and behavioral (movement time, error rate, and skill) outcome measures. Our findings showed no significant additional effects in implicit motor sequence learning in the trained hand following focal stimulation of a-tDCS over any of the FPN superficial sites compared to sham group. Transfer of learning into the untrained hand were only observed for movement time not for error rate or skill in all different stimulation sites. We also found no significant effects on CSE, SICI, and ICF in M1 area following intervention. There are some possible reasons behind the negative results.

One explanation can be related to the a-tDCS characteristics used in this study. Because we aimed to selectively stimulate M1, not nearby areas, such as premotor cortex, supplementary motor area or primary sensory area, we used a small electrode size of 3 cm^2^ in order to adjust the size of the electrode, low intensity stimulation of 0.3 mA was used that produced a current density of 0.1 mA/cm^2^. However, some studies have shown that a small electrode size (3 cm^2^) or current density (0.1 mA/cm^2^) can affect M1 excitability at rest state (Nitsche and Paulus, 2000; Bastani and Jaberzadeh, 2013a; Vaseghi et al., 2015a), we observed no changes in CSE after application a-tDCS during SVIPT. Regarding the issue that performing a cognitive or motor task during stimulation can modulate the effects of tDCS on M1 excitability (Antal et al., 2007), it is likely a-tDCS with these characteristics, when applied during training, do not impact on neurophysiologic outcomes.

In addition, our results indicated no changes in the excitability of M1 following a single session of a-tDCS over DLPFC or PPC combined with training of SVIPT. Even though, cortical excitability of DLPFC or PPC were not assessed in the current study but we can assume no changes in excitability of DLPFC and PPC following a-tDCS with small-size stimulating electrodes of these sites. This may be considered as a reason behind the absence of a-tDCS effects on the performed task. We also found no significant changes in SICI and ICF of M1 after stimulation, which is consistent with a systematic review that showed tDCS generates little-to-no neurophysiological effects on SICI or ICF (Horvath et al., 2015). However, a significant enhancement in SICI and ICF in M1 area reported in a study by Rivera-Urbina et al. (2015) after application of a-tDCS over PPC at rest state, not concurrently with training (Rivera-Urbina et al., 2015). In line with our findings Uehara et al. (2013) found no direct DLPFC-M1 connectivity during performance of a rhythmic of abduction with the index finger (Uehara et al., 2013).

Although we expected single-session focal stimulation a-tDCS over DLPFC or PPC led to enhance sequential learning, compared to the sham group, due to neuropsychological evidence strongly supports the role of PPC or DLPFC in higher cognitive functions or sensorimotor integration (Bahrick et al., 1954; Seger, 1994; Castro-Alamancos et al., 1995; Castro-Alamancos and Connors, 1996), no specific effects were found on SVIPT. The absence of any effects for DLPFC or PPC a-tDCS in the current study can be explained by tDCS characteristics or task-dependent effects of a-tDCS on learning and memory formation (Saucedo Marquez et al., 2013). A mini review by Ammann et al. (2016) showed that the standard tDCS montage (the current intensity (1-2 mA) and electrode size (25–35 cm^2^) on different areas of the brain can lead to significant positive results on motor learning (Ammann et al., 2016). some studies have shown a-tDCS of the left DLPFC (with a range of current density from 0.028 to 0.1 mA/cm^2^ and electrode sizes of 25–35 cm^2^) could modify different kinds of tasks, such as implicit probabilistic classification learning (Kincses et al., 2004), sequential-letter memory tasks (Fregni et al., 2005), cognitive tasks (Kuo and Nitsche, 2015) as well as mental practice (Foerster et al., 2013). In spite of that, in line with the findings in the current study, literature also indicates that even utilization of standard intensity and electrode size is not sufficient to improve sensorimotor learning of a highly skilled tasks with a single session application in healthy participants (Butefisch et al., 2000; Boggio et al., 2006; Ni et al., 2009; Saiote et al., 2013; Minarik et al., 2015; Hashemirad et al., 2016). In line with our results a study by Convento et al. (2014) showed no improvement in performance of a Jebsen–Taylor Hand Function Test, after single-session left PPC with electrode size of 5 cm ×5 cm and intensity of 2 mA (Convento et al., 2014).

Another possible reason can explain our null results is that ceiling effects may be present in healthy and young participants. In addition, inter variability between participants (Lopez-Alonso et al., 2014) might be another reason for negative results obtained in the current study. Regarding to the huge controversy in the results of tDCS studies, further research is needed to compare the effects of different protocols of tDCS in terms of intensity, electrode size as well as stimulation sites on improvement of motor learning in different kinds of motor tasks.

Our results demonstrated that transfer of learning into the untrained hand only occurred for movement time not for the error rate or skill. Contrary to our results, Camus et al. (2009) found transfer learning into the left untrained hand in both movement time and error rate after six blocks of training using SVIPT with the right hand (Camus et al., 2009). There are several factors that may be responsible for this discrepancy. They probably used explicit types of SVIPT due to the number of target forces and feedback was given throughout their experiment, while participants learned SVIPT implicitly in our experiment. In addition, they did not apply a-tDCS during training.

Although we found no between-groups effects following the single-session a-tDCS over the FPN superficial sites, further research is need to find out what specific cortical site is involved in sequence learning as well as transfer learning into the opposite site for a precision control task, such as SVIPT. It should be noted that the method used in the literature for assessment of behavioral outcomes in SVIPT is trial-based. In this method, behavioral outcomes are measured in the span of a trial. This method of data handling is gross and does not able to detect detailed changes which might occurred in each target force at early stage of learning during SVIPT. So, further research is needed to investigate tDCS effects within the span of an individual force (this is the subject of a forthcoming publication). Increasing our knowledge about sequence learning, especially for fine control tasks may have significant implications for rehabilitation of patients who are suffered from neurological disorders, such as a stroke or Parkinson’s disease.

### Limitations and Suggestions

There are some limitations in this study. We included healthy young individual participants so we cannot extrapolate our results to elderly or patient’ populations. Regarding to the lack of effects of a-tDCS on cortical outcomes (CSE, ICF, and SICI), one possible reason for the null findings may be related to the small size of the stimulating electrodes. Further research using larger electrode sizes over the FPN sites is needed to investigate the possible excitatory effects of nearby cortical sites on cortical and behavioral outcomes during a fine motor sequence task such as SVIPT.

We assessed outcome measures only one day after intervention, and long term effects of a-tDCS on behavioral outcome measures were not demonstrated in this study. In the current study, we used a-tDCS and TMS for finding functional connectivity of FPN sites; using new techniques such as double-coil TMS and diffusion tensor imaging (DTI) can be more helpful to find out functional connectivity and specific roles of the FPN sites in motor sequence leaning. We also measured general behavioral outcomes including movement time, error rate and skill in the level of each trial; the measurement of other variables such as reaction time or force deviations in the level of each target force might be more sensitive to motor sequence learning and induced plasticity following intervention such as tDCS.

## Conclusion

Our results demonstrated that a single session a-tDCS with small-size stimulating electrodes over DLPFC, M1, or PPC combined with training of SVIPT has no significant additional effects on implicit motor sequence learning in the trained hand. We also found no significant changes in M1 excitability, inhibition or facilitation following a-tDCS during SVIPT. Additionally, transfer learning into the untrained hand was seen only for speed but not for accuracy or skill after application of a-tDCS during a fine control task such as SVIPT.

## Author Contributions

The present article is a part of PhD thesis of the corresponding author FH. The main supervisor SJ and the co-supervisors, PF and MZ, helped the corresponding author to develop the study design.

## Conflict of Interest Statement

The authors declare that the research was conducted in the absence of any commercial or financial relationships that could be construed as a potential conflict of interest.
