# Supplementary material for: Single-Session Anodal tDCS with Small-Size Stimulating Electrodes Over Frontoparietal Superficial Sites Does Not Affect Motor Sequence Learning
Source: Front Hum Neurosci. 2017 Apr 3;11:153. doi: 10.3389/fnhum.2017.00153 (PMC5376552; doi:10.3389/fnhum.2017.00153)
Supplement: Supplementary file 1 [file Data_Sheet_1.docx]

Table S1: The results of ANOVA to determine the side effects of a-tDCS during application of a-tDCS among the four experimental conditions. Data are presented as mean ± SEM.

| tDCS side effects | Time | **Active electrode** | | | | | **Return electrode** | | | | |
| --- | --- | --- | --- | --- | --- | --- | --- | --- | --- | --- | --- |
|  |  | M1 | DLPFC | PPC | Sham | p-value | M1 | DLPFC | PPC | Sham | p-value |
| Numbness | 5 min | 0.16±0.5 | 0 | 0 | 0 | .402 | 0 | 0 | 0 | 0 | - |
|  | 10 min | 0 | 0 | 0 | 0 | - | 0 | 0 | 0 | 0 | - |
|  | 15 min | 0 | 0 | 0 | 0 | - | 0 | 0 | 0 | 0 | - |
|  | 20 min | 0 | 0 | 0 | 0 | - | 0 | 0 | 0 | 0 | - |
|  | 5 min | 1.5±2.6 | 1.5±2.1 | 0.75±1.7 | 0.33±1.1 | .392 | 0 | 0.3±1.15 | 0 | 0 | .402 |
| Itching | 10 min | 1.5±2.4 | 0.83±1.2 | 1±1.5 | 0 | .111 | 0 | 0.33±1.15 | 0 | 0 | .402 |
|  | 15 min | 1.08±1.7 | 0.83±1.5 | 1.1±1.9 | 0 | .236 | 0 | 0.08±0.28 | 0 | 0 | .72 |
|  | 20 min | 1.7±2.5 | 0.66±1.6 | 1.08±2.1 | 0 | .143 | 0 | 0.83±0.28 | 0 | 0 | .402 |
| Burning | 5 min | 1.2±2.4 | 1.3±2.8 | 0.5±1.08 | 0 | .31 | 0.5±1.2 | 0.75±1.5 | 0.58±1.5 | 0.16±0.5 | .72 |
|  | 10 min | 1.5±2.9 | 0.9±2.2 | 0.5±1.2 | 0 | .259 | 0.33±0.88 | 0.33±0.88 | 0.41±1.4 | 0 | .72 |
|  | 15 min | 1.6±3.02 | 0.75±1.5 | 0.16±0.57 | 0 | .091 | 0.16±0.57 | 0.33±0.65 | 0.16±0.57 | 0 | .81 |
|  | 20 min | 1.16±2.4 | 0.37±1.1 | 0.58±1.5 | 0 | .331 | 0.16±0.57 | 0.083±0.28 | 0.16±0.57 | 0 | .74 |
| Pain | 5 min | 0 | 0 | 0.3±1.15 | 0 | .402 | 0 | 0 | 0 | 0 | - |
|  | 10 min | 0 | 0 | 0 | 0 | - | 0.16±0.57 | 0 | 0 | 0 | .402 |
|  | 15 min | 0 | 0 | 0 | 0 | - | 0.33±1.1 | 0 | 0 | 0 | .402 |
|  | 20 min | 0 | 0 | 0.16±0.57 | 0 | .402 | 0.33±1.1 | 0.083±0.28 | 0 | 0 | .47 |
| Nervousness | 5 min | 0.4±1.4 | 0 | 0 | 0 | .402 | 0 | 0 | 0 | 0 | - |
|  | 10 min | 0.4±1.4 | 0 | 0 | 0 | 0.402 | 0 | 0 | 0 | 0.25±0.86 | .402 |
|  | 15 min | 0.4±1.4 | 0 | 0 | 0 | .402 | 0 | 0 | 0 | 0.25±0.86 | .577 |
|  | 20 min | 0.4±1.4 | 0 | 0 | 0 | .402 | 0 | 0 | 0 | 0.25±0.86 | .577 |
| Headache | 5 min | 0 | 0 | 0 | 0 | - | 0 | 0 | 0 | 0 | - |
|  | 10 min | 0 | 0 | 0 | 0 | - | 0 | 0 | 0 | 0.25±0.86 | .402 |
|  | 15 min | 0 | 0 | 0 | 0 | - | 0 | 0 | 0 | 0.33±1.15 | .402 |
|  | 20 min | 0 | 0 | 0 | 0 | - | 0 | 0 | 0 | 0.33±1.15 | .402 |

Table S2: The results of Pearson correlations between the ratios of cortical and behavioral outcomes in two experimental sessions.

| Experimental sessions  ( N= 48 ) | Cortical / behavioral outcomes | Ratio movement time | | Ratio error rate | | Ratio skill | |
| --- | --- | --- | --- | --- | --- | --- | --- |
|  |  | r | p | r | p | r | p |
| Session 1  (post 15 min/baseline) | Ratio MEPs | -.11 | .44 | -.027 | .85 | .051 | .73 |
|  | Ratio SICI | .035 | .81 | -.12 | .41 | .074 | .61 |
|  | Ratio ICF | -.21 | .14 | -.21 | .13 | .21 | .14 |
| Session 2  (post 24 hours/baseline) | Ratio MEPs | -.056 | .75 | .086 | .56 | -.14 | .32 |
|  | Ratio SICI | -.13 | .36 | -.087 | .55 | -.18 | .22 |
|  | Ratio ICF | **-.41**** | .003 | -.055 | .71 | -.17 | .24 |
